# A threshold of β-CTX (0.3 ng/mL) with low estradiol identifies high-risk perimenopausal women for bone loss: a cross-sectional study

**DOI:** 10.3389/fendo.2025.1709858

**Published:** 2025-11-20

**Authors:** Xufen Feng, Wenjun Xiao, Rongshan Zhang

**Affiliations:** 1 Department of Laboratory Medicine, Ganzhou Maternal and Child Health Care Hospital, Ganzhou, Jiangxi, China; 2 Department of Laboratory Medicine, Ganzhou People’s Hospital, Ganzhou, Jiangxi, China

**Keywords:** β-C-terminal telopeptide, estradiol, bone loss, perimenopausal, prediction

## Abstract

**Background:**

Increasing evidence has demonstrated accelerated bone loss during perimenopause. The detection of bone loss relies heavily on dual-energy X-ray absorptiometry (DXA). However, DXA is not sensitive enough for early bone loss. Therefore, an easy and sensitive method is urgently needed for identifying high-risk women before irreversible bone loss occurs.

**Objective:**

To 1) define a clinically meaningful β-CTX threshold (≥ 0.3 ng/mL) for perimenopausal bone loss prediction, 2) assess the predictive value of E2 and β-CTX, both individually and in combination, for bone loss in perimenopausal women.

**Methods:**

One hundred and thirty female participants met the inclusion/exclusion criteria were enrolled in this study from March 2024 to March 2025. Enrolled subjects underwent DXA examination and blood tests, including measurements of E2, β-CTX, TP1NP, D3, and IGF-1. The correlations between E2, β-CTX, TP1NP, D3, IGF-1 and T-scores were performed using Spearman correlation analysis. The predicting value of E2, β-CTX and combination for perimenopausal bone loss were studied by ROC curve analysis.

**Results:**

There were significant correlations between E2, β-CTX, TP1NP and T-scores, but not between D3, IGF-1 and T-scores. The threshold value of E2 alone in predicting perimenopausal bone loss was 62.7 pmol/L. Its sensitivity and specificity were 79.1% and 93.2%, respectively. The threshold value of β-CTX alone in predicting perimenopausal bone loss was 0.30 ng/mL. Its sensitivity and specificity were 79.3% and 96.4%, respectively. The ROC curve of E2 combined with β-CTX showed that the AUC was 0.950. Its sensitivity and specificity were 88.4% and 97.7%, respectively, which were higher than that in E2 and β-CTX alone.

**Conclusion:**

A clinically meaningful β-CTX threshold (≥ 0.3 ng/mL) was defined for perimenopausal bone loss prediction, and the combination of E2 and β-CTX is a simple and reliable method for predicting perimenopausal bone loss, with high sensitivity and specificity. A threshold of β-CTX (0.3 ng/mL) with low estradiol identifies high-risk perimenopausal women for bone loss.

## 1 Introduction

Osteoporosis, a systemic skeletal disorder characterized by compromised bone strength and increased fracture risk, represents a major public health burden, particularly among aging women (1). Globally, over 200 million women suffer from osteoporosis, with postmenopausal women accounting for 80% of cases (2). While extensive research has focused on postmenopausal bone loss, the perimenopausal transition, a critical window of hormonal fluctuation preceding menopause, remains understudied, despite increasing evidence demonstrated that accelerated bone loss begins during this phase or earlier (3). During perimenopause, estrogen levels, particularly estradiol (E2), decline erratically, leading to uncoupled bone remodeling where bone resorption outpaces formation (4). This imbalance further results in bone mineral density (BMD) reduction, yet clinical detection often occurs too late for effective intervention.

To the best of our knowledge, the detection of bone loss relies heavily on dual-energy X-ray absorptiometry (DXA) to measure BMD (5). However, DXA has several limitations in clinical practice. First, DXA is not sensitive enough for early bone loss, because it detects bone loss only after 20-30% of trabecular bone has already deteriorated (6). Moreover, DXA is costly, lacks portability, and is inaccessible in many low-resource settings. Besides, the detection of bone loss required repeated DXA measurements, which may expose patients to excessive radiation doses. These highlight the urgent need for a simple and sensitive method to identify high-risk women before irreversible bone loss occurs.

In recent years, growing evidence has demonstrated the significance of serum estradiol (E2) and bone turnover markers in the diagnosis of postmenopausal bone loss and osteoporosis (7). Estradiol plays a pivotal role in maintaining bone homeostasis by suppressing osteoclast activity (7, 8). As E2 levels drop during perimenopause, osteoclast-mediated bone resorption surges, releasing type I collagen degradation products such as β-C-terminal telopeptide (β-CTX) into circulation (9). Serum β-CTX, a sensitive marker of bone resorption, rises significantly during early menopause and correlates strongly with future fracture risk (9). Existing studies have predominantly investigated serum estradiol and bone turnover markers in postmenopausal cohorts, leaving its diagnostic thresholds and predictive power in perimenopausal women poorly defined (10).

Low E2 alone usually indicates higher osteoclast activity, and elevated β-CTX reflects resorption intensity (11). This synergistic relationship aligns with the “coupling” theory of bone remodeling. However, no studies have yet explored whether combining E2 and β-CTX improves early bone loss prediction in perimenopausal women.

Therefore, this present study aimed to 1) define a clinically meaningful β-CTX threshold (≥ 0.3 ng/mL) for perimenopausal bone loss prediction; 2) evaluate the predictive value of E2 and β-CTX, both individually and in combination, for bone loss in perimenopausal women. By validating the E2-β-CTX axis as a dual-marker tool, the study proposed a simple and sensitive method for identifying high-risk perimenopausal women, addressing the limitations of current guidelines and enabling timely interventions to prevent further bone loss in this vulnerable population.

## 2 Methods

### 2.1 Study design and participants

This is a cross-sectional study. One hundred and thirty consecutive participants were recruited in this study from March 2024 to March 2025. The inclusion criteria were as follow: (1) Female; (2) Age from 40–60 years. The exclusion criteria were as follow: (1) Diagnosed with secondary osteoporosis; (2) Undergoing anti-osteoporosis therapies; (3) Participants with hyperthyroidism, parathyroid disorders, diabetes mellitus, osteoarthropathy, or metabolic bone diseases; (4) Participants with ovarian or uterine lesions; (5) Participants with a menopausal duration exceeding one year; (6) Participants with acute or chronic insufficiency of heart, lung, liver, kidney, or other organs; (7) Participants with autoimmune diseases; (8) Participants with malignant tumors; (9) Participants with psychiatric disorders (e.g., dementia, psychosis) or those unable to cooperate with the study; (10) Participants using oral anticonceptives or hormone replacement therapy.

### 2.2 Blood examination

Enrolled subjects underwent blood tests, including measurements of Estradiol (E2) (pmol/L), β-CTX (ng/mL), Total Procollagen Type I N-terminal Propeptide (TP1NP) (ng/mL), Cholecalciferol (D3) (ng/mL), and Insulin-like Growth Factor 1 (IGF-1) (ng/mL). Specifically, after fasting for 12 hours, venous blood was collected from subjects in a seated position the following morning under fasting conditions. Post-collection, blood samples were centrifuged at 3,500 rpm for 15 minutes (radius: 8 cm), and the resulting serum was separated and stored in an ultra-low-temperature freezer at -80 °C. Serum levels of E2, β-CTX, TP1NP, D3, and IGF-1 were quantified using Electrochemiluminescence method (Roche cobasE801/E601). All the reagents were purchased from Roche Diagnostics CmbH (Germany). Each biomarker was tested in triplicate, with a Coefficient of Variation (CV) (standard deviation (SD)/Mean × 100%) requirement of ≤5%, and the average value was recorded for analysis. All blood samples were centrifuged within a maximum of 30 minutes after collection to ensure biomarker stability. All centrifugation steps were performed using the same centrifuge model to maintain processing consistency across all samples.

### 2.3 DXA examination

Enrolled subjects underwent DXA examination (12). The DXA devices used in this study was manufactured by GE Healthcare (USA) (Model: DPX-NT). Specifically, the patient lies supine on a padded table while a scanner passes over specific regions (e.g., lumbar spine, femur, or forearm). Low-dose X-rays are emitted, and differential absorption by bone and soft tissue is analyzed to calculate BMD. Results are expressed as T-scores (comparison to young adult peak bone mass). Each subject was tested three times, with CVs requirement of ≤3%, and the average T-scores was recorded. The average -1 ≤ T*-*scores indicates normal bone mass; -2.5 < T-scores < -1 signifies osteopenia (low bone mass); and -2.5 ≤ T-scores indicates osteoporosis. In this study, participants with T-scores < -1 were defined as bone loss, and participants with -1 < T-scores was defined as normal without bone loss.

### 2.4 Statistical analysis

The data in this study was analyzed using SPSS (Version 25.0) and Graphpad Prism (Version 9.4.1). The χ2 test, t-test, and rank-sum test were used depending on the data type. The Spearman correlation coefficient was used to evaluate the correlation between E2 and BMD, between β-CTX and BMD. Potential confounding factors, including age, body mass index (BMI), hypertension, and hyperlipidemia, were evaluated for significant differences between groups before correlation analyses. Since these variables did not differ significantly (*p* > 0.05), Spearman correlation analysis was used for simplicity. In addition, partial correlation analyses adjusting for age and BMI were conducted to confirm the robustness of the associations between D3, IGF-1, and T-scores. The adjusted results were consistent with the unadjusted findings, showing no statistically significant correlations. Receiver Operating Characteristic (ROC) curves analysis were utilized to assess the diagnostic performance of E2, β-CTX, and their combination in detecting bone loss. Categorical data are presented as frequencies or percentages, and normally distributed continuous data are expressed as mean ± standard deviation (SD). A *P* value less than 0.05 was considered statistically significant.

## 3 Results

### 3.1 Demographic characteristics of included participants

One hundred and thirty female participants met the inclusion/exclusion criteria were enrolled in this study, with an average age of 49.88 ± 5.59 years. The subjects were divided into two groups based on T-score, namely Normal group (-1 ≤ T-scores) and Bone Loss (BL) group (T-scores < -1). There were 44 subjects in the normal group, with an average age of 49.64 ± 3.57 years, and 86 participants in the BL group, with an average age of 50.13 ± 5.32 years (**Table 1**). No significant differences were found in the average age between the normal and BL groups (*p* = 0.281). The average T scores in the normal group (- 0.11 ± 0.47) was significantly higher than that in the BL group (-2.39 ± 0.50) (*p* < 0.0001). However, there were no significant differences in the Body Mass Index (BMI), hypertension and hyperlipidemia between the normal and BL groups (*p* = 0.724, *p* = 0.715, *p* = 0.783, respectively).

**Table 1:** The demographic characteristics of included participants.

Variables	Normal group	BL group	*P* value
Number	44	86	NA
Average age (years)	49.64 ± 3.57	50.13 ± 5.32	0.281
T scores	-0.11 ± 0.47	-2.39 ± 0.50	< 0.0001
BMI (kg/m^2^)	22.48 ± 2.68	22.69 ± 3.02	0.724
Hypertension	9/44	20/86	0.715
Hyperlipidemia	6/44	13/86	0.783

*BMI, Body Mass Index; NA, Not applicable.

### 3.2 Comparison of blood tests

The levels of E2, β-CTX, TP1NP, D3, and IGF-1 in blood plasma were detected and analyzed. The results were shown in **Table 2**. The levels of E2 were 420.91 ± 259.21 pmol/L in the normal and 77.10 ± 186.10 pmol/L in the BL group, respectively. The level of E2 in the normal group was significantly higher than that in the BL group (*p* < 0.0001), indicating that the decrease of E2 levels may contributes to bone loss in perimenopausal women. Furthermore, the levels of β-CTX, TP1NP in the normal group were significantly lower than that in the BL group (*p* < 0.0001, *p* < 0.0001, respectively). However, even though the levels of D3, and IGF-1 in the normal group were lower than that in the BL group, no significant differences were found (*p* = 0.2806, *p* = 0.3118, respectively). These results indicated that although D3 and IGF-1 are commonly used biomarkers for bone metabolism, they may not be very accurate in predicting bone loss compared to β-CTX and TP1NP.

**Table 2:** Comparison of blood test.

Variables	Normal group	BL group	*P* value
E2 (pmol/L)	420.91 ± 259.21	77.10 ± 186.10	< 0.0001
β-CTX (ng/mL)	0.20 ± 0.06	0.46 ± 0.20	< 0.0001
TP1NP (ng/mL)	42.13 ± 15.21	58.74 ± 21.86	< 0.0001
D3 (ng/mL)	23.88 ± 8.85	25.66 ± 8.87	0.2806
IGF-1 (ng/mL)	106.30 ± 32.21	112.90 ± 39.79	0.3118

*E2, Estradiol; β-CTX, β-C-terminal telopeptide; TP1NP, Total Procollagen Type I N-terminal Propeptide; D3, Cholecalciferol; IGF-1, Insulin-like Growth Factor 1.

### 3.3 Correlation analysis between E2, β-CTX, TP1NP, D3, IGF-1 and T scores

**Figure 1:**
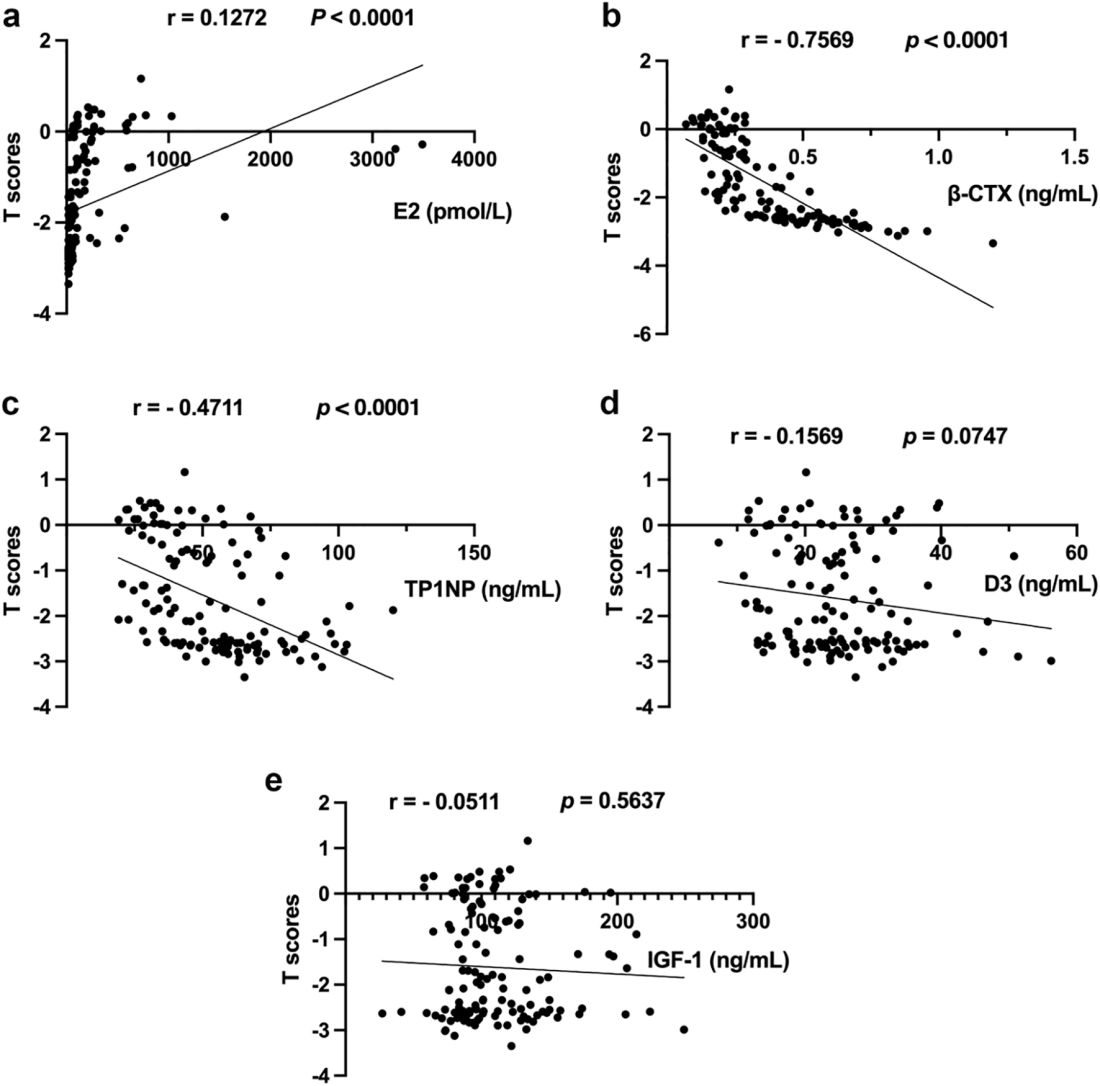
The results of correlation analysis. **(a)** Correlation analysis between E2 and T scores; **(b)** Correlation analysis between β-CTX and T scores; **(c)** Correlation analysis between TP1NP and T scores; **(d)** Correlation analysis between D3 and T scores; **(e)** Correlation analysis between IGF-1 and T scores.

Subsequently, the correlation between E2, β-CTX, TP1NP, D3, IGF-1 and T scores were performed. The **Figure 1a** shows a positive correlation between E2 levels and T-scores (*p* < 0.0001), indicating that the lower the estrogen levels, the higher the risk of bone loss, though the Pearson correlation coefficient (r = 0.1272) was low. Furthermore, **Figures 1b, c** showed that the levels of β-CTX and TP1NP showed a negative correlation with T-scores (r = - 0.7569, *p* < 0.0001; r = - 0.4711, *p* < 0.0001, respectively). However, even though the levels of D3 and IGF-1 showed negative correlation with T-scores (**Figures 1d, e**), there was no statistical significance (*p* = 0.0747, *p* = 0.5637, respectively) and the Pearson correlation coefficient (r = - 0.1569, r = - 0.0511, respectively) were low. These findings further confirmed that the levels of D3 and IGF-1 may be not appropriate for predicting bone loss in perimenopausal women.

### 3.4 The predictive value of E2 and β-CTX alone versus in combination for bone loss

**Figure 2:**
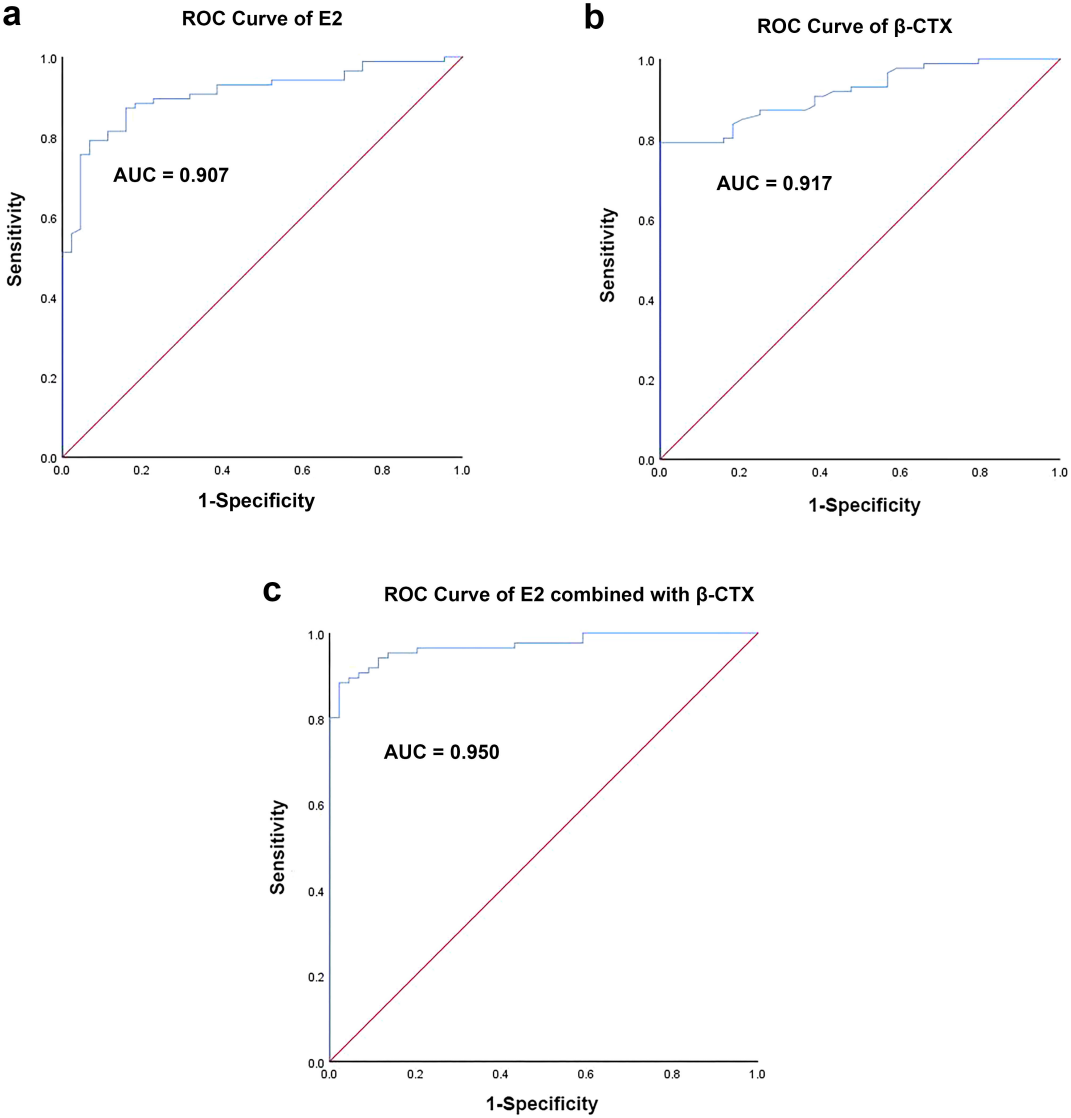
The results of ROC curves analysis. **(a)** ROC curve of E2; **(b)** ROC curve of β-CTX; **(c)** ROC curve of E2 combined with β-CTX.

ROC curves analysis was utilized to assess the diagnostic performance of E2, β-CTX, and their combination in detecting bone loss in perimenopausal women. The AUC of E2 shown in **Figure 2a** was 0.907. The threshold value of E2 in predicting perimenopausal bone loss was 62.7 pmol/L, which indicated that perimenopausal women with plasma estradiol concentrations below 62.7 pmol/L may suffer bone loss (**Table 3**). Its sensitivity and specificity were 79.1% and 93.2%, respectively (*p* < 0.001, **Table 3**). Moreover, **Figure 2b** showed the ROC curve of β-CTX and the AUC was 0.917. The threshold value of β-CTX in predicting perimenopausal bone loss was 0.30 ng/mL. Its sensitivity and specificity were 79.3% and 96.4%, respectively (*p* < 0.001, **Table 3**). The sensitivity of both biomarkers in predicting bone loss is comparable, but β-CTX exhibits superior specificity to E2. Additionally, **Figure 2c** showed the ROC curve of E2 combined with β-CTX and the AUC was 0.950. Its sensitivity and specificity were 88.4% and 97.7%, respectively, which were higher than that in E2 and β-CTX alone (*p* < 0.001, **Table 3**).

**Table 3:** The predictive value of E2 and β-CTX alone versus in combination for bone loss.

Variables	Sensitivity	Specificity	AUC	95% CI	Threshold value	*P* Value
E2	79.1%	93.2%	0.907	(0.856, 0.958)	62.7	< 0.001
β-CTX	79.3%	96.4%	0.917	(0.871, 0.962)	0.30	< 0.001
E2-β-CTX	88.4%	97.7%	0.950	(0.925, 0.975)	/	< 0.001

*E2, Estradiol; β-CTX, β-C-terminal telopeptide; CI, Confidence Intervals.

## 4 Discussion

The perimenopausal stage of life marks the gradual decline in a woman’s fertility, accompanied by the progressive deterioration of ovarian function (13). Bone loss is a significant complication during this period, which can lead to osteoporosis and even fractures in severe cases (4, 14). Currently, BMD measurement is the primary method for assessing bone loss. DXA is widely used to measure hip and spine BMD, accurately reflecting changes in bone mass and serving as the gold standard for osteoporosis (OP) diagnosis (5). However, since BMD measurement is based on two-dimensional imaging, it cannot precisely capture the complex three-dimensional bone structure, potentially leading to insufficiently accurate OP risk assessment. Moreover, the rate of bone loss varies among perimenopausal women, and the speed of BMD decline differs from person to person. Some patients may experience rapid bone loss in the early stages of menopause, while others may undergo a more gradual reduction in bone mass (15). Therefore, a single-time-point BMD measurement may not accurately reflect the bone condition of all individuals, especially for women with faster bone loss rates. However, repeated BMD measurements may expose patients to excessive radiation doses. Therefore, exploring simpler, more reliable, and more sensitive detection methods is of great significance for evaluating bone loss in perimenopausal women. In this study, we found that a combination of E2 and β-CTX is sensitive and reliable method to predict bone loss in perimenopausal women. Specifically, a threshold of β-CTX (0.3 ng/mL) with low E2 identified high-risk perimenopausal women for bone loss.

In this study, the level of E2 in the normal group was significantly higher than that in the BL group, and the level of E2 shows a positive correlation with T-scores. This is likely because estrogen exerts crucial protective effects on bone metabolism (16). It helps maintain bone mass by reducing bone resorption and enhancing bone formation. Conversely, when estrogen levels become insufficient, bone resorption increases while bone formation decreases, eventually leading to bone loss. It is noteworthy that the Pearson correlation coefficient (r = 0.1272) between E2 and T-scores was low. This may stem from the non-linear nature of the relationship between estradiol and bone mineral density, potentially involving either a threshold effect (where bone loss accelerates below critical concentrations) or a saturation effect (where protective benefits plateau beyond certain levels). Furthermore, circulating E2 exhibits dynamic fluctuations throughout the menstrual cycle, rendering single random measurements potentially inadequate for reflecting physiologically relevant concentrations (17). The subsequently ROC curve analysis verified this speculation, which showed that the threshold value of E2 was 62.7 pmol/L, and the sensitivity and specificity were 79.1% and 93.2%, respectively. These findings also demonstrate the limited predictive value of E2 alone for assessing perimenopausal bone loss, highlighting the necessity of adopting dual- or multi-marker prediction approaches.

β-CTX serves as a specific biomarker for bone collagen breakdown (18). Elevated serum concentrations of this marker typically indicate heightened osteoclast-mediated bone resorption activity. In this study, the level of β-CTX in the BL group was significantly higher than that in the normal group. This is because that in perimenopausal women experiencing significant estrogen decline, the loss of estrogen’s inhibitory effect on osteoclasts reduces the suppression of bone collagen degradation. Consequently, these triggers accelerated bone resorption, ultimately leading to distinctly elevated β-CTX levels (11). Furthermore, the levels of β-CTX showed a strongly negative correlation with T-scores, which was consistent with previous studies (11). Although several studies have demonstrated the negative correlation between β-CTX and bone loss, the optimal threshold value of β-CTX remains unknown. In the ROC curve analysis, we found that the optimal threshold value of β-CTX was 0.30 ng/mL, with high sensitivity (79.3%) and specificity (96.4%).

TP1NP serves as a biochemical marker reflecting osteoblastic activity (19). Elevated serum levels typically indicate increased collagen synthesis and heightened bone formation. However, in this study, we observed a paradoxical TP1NP elevation in the bone loss group. This can be attributed to a compensatory response to excessive bone loss. Specifically, in perimenopausal women experiencing estrogen decline, this hormonal shift not only accelerates bone resorption but also impacts bone formation dynamics. Under certain conditions, these individuals may exhibit an elevation in bone formation as a compensatory response to excessive bone loss, which can subsequently drive up TP1NP concentrations (20). Elevated TP1NP is thus a marker of this active but imbalanced bone formation. This result also suggests that TP1NP alone has limited predictive value for bone loss. Furthermore, although there was a negative correlation between TP1NP and T-scores, but the Pearson correlation coefficient was lower compared with β-CTX, which also was one of the reasons why we chose a combination of E2 and β-CTX but not E2 and TP1NP.

D3 and IGF-1 represent two pivotal bioactive molecules that play critical regulatory roles in bone metabolism (21). Moreover, their respective plasma concentrations can serve as indirect yet clinically valuable biomarkers of bone turnover status. In this study, no significant differences were found between normal and BL group in the concentrations of D3 and IGF-1. Furthermore, to the best of our knowledge, the relationship between D3, IGF-1 and T-scores remains controversial, though both D3 and IGF-1 were demonstrated to promote bone formation (19, 22). In this study, there is no significant correlation between D3 and T-scores, and between IGF-1 and T-scores. The lack of significant D3 and IGF-1 correlations with T-scores in our study may reflect several factors. First, the perimenopausal stage is characterized by complex endocrine changes dominated by estrogen decline, which might overshadow the bone effects of D3 and IGF-1. Second, most of our participants showed relatively adequate vitamin D and nutritional status, reducing variability and limiting the ability to detect associations. Third, as BMD was measured by DXA, which mainly reflects bone mineral content but not trabecular microarchitecture, potential microstructural benefits of D3 or IGF-1 may have been underestimated. Finally, circulating IGF-1 levels are influenced by hepatic production and binding proteins, which may not directly reflect local IGF-1 activity in bone tissue. Moreover, to minimize potential bias from confounding variables such as age and BMI, partial correlation analyses were performed, which confirmed that D3 and IGF-1 were still not significantly correlated with T-scores after adjustment. This suggests that the absence of correlation was not driven by these demographic factors but may instead reflect their limited independent contribution to bone density in perimenopausal women.

Additionally, in this study, ROC analysis showed that the AUC of dual-biomarker (E2 combined with β-CTX) was 0.950. Its sensitivity and specificity were 88.4% and 97.7%, respectively, which were higher than that in E2 and β-CTX alone. These indicate that the combination of E2 and β-CTX is a simple and reliable method for predicting perimenopausal bone loss, with high sensitivity and specificity. E2 regulating bone formation via osteoblast activity and β-CTX reflecting osteoclast-mediated resorption. This synergistic relationship aligns with the “coupling” theory of bone remodeling, which may be the theoretical foundation of the excellent results. However, the AUC of 0.950 for the combined markers suggests excellent discriminative ability. This may be due to the limited sample size and other uncontrollable variables, and further study is required to verify the results.

This study has several notable limitations. First, although our sample size (n=130) provided adequate statistical power for detecting strong correlations and predictive performance, the relatively small and single-center cohort may limit the generalizability of the findings. The homogeneity of the participants in terms of geography and ethnicity may introduce selection bias. Therefore, larger multi-center studies involving more diverse populations are necessary to verify whether the proposed thresholds of E2 (<62.7 pmol/L) and β-CTX (≥0.30 ng/mL) are applicable across broader perimenopausal populations. Additionally, longitudinal follow-up studies could further validate the predictive stability of these biomarkers over time. Second, the associations between vitamin D3/IGF-1 concentrations and T scores warrant further confirmation through rigorously designed studies. Third, this is cross-sectional study, without any interventions and longitudinal data that would better support the proposed predictive thresholds. Future studies with long-term follow-up or additional interventions are needed to further validate our findings. Four, a single random measurement of E2 may introduce potential bias, and incorporating repeated measurements or standardized sampling timing to minimize such potential bias was proposed in the future studies. Additionally, we had to acknowledge potential optimism due to the cross-sectional design and lack of external validation.

## 5 Conclusion

A clinically meaningful β-CTX threshold (≥ 0.3 ng/mL) was defined for perimenopausal bone loss prediction, and the combination of E2 and β-CTX is a simple and reliable method for predicting perimenopausal bone loss, with high sensitivity and specificity. Specifically, a threshold of β-CTX (0.3 ng/mL) with low estradiol identifies high-risk perimenopausal women for bone loss.

This dual-indicator prediction strategy can not only serve as an effective alternative to DXA for BMD measurement, avoiding repeated radiation exposure, but also act as a cost-efficient and high-performance screening tool for identifying high-risk perimenopausal women before irreversible bone loss occurs in clinical practice when there is DXA lacking.

## Data Availability

The raw data supporting the conclusions of this article will be made available by the authors, without undue reservation.
