# Correction: A threshold of β-CTX (0.3 ng/mL) with low estradiol identifies high-risk perimenopausal women for bone loss: a cross-sectional study

**DOI:** 10.3389/fendo.2025.1756303

**Published:** 2025-12-08

**Authors:** Xufen Feng, Wenjun Xiao, Rongshan Zhang

**Affiliations:** 1 Department of Laboratory Medicine, Ganzhou Maternal and Child Health Care Hospital, Ganzhou, Jiangxi, China; 2 Department of Laboratory Medicine, Ganzhou People’s Hospital, Ganzhou, Jiangxi, China

**Keywords:** β-C-terminal telopeptide, estradiol, bone loss, perimenopausal, prediction

In the published article, there was a mistake in the **Funding** statement. The **Funding** statement for the Science and Technology Plan of the Health Commission of Jiangxi Province was displayed as “SKJP220233805”. The correct statement is “Science and Technology Plan of the Health Commission of Jiangxi Province (No. 202410824).’’

The original version of this article has been updated.

